# Renoprotective potential of concomittant medications with SGLT2 inhibitors and renin-angiotensin system inhibitors in diabetic nephropathy without albuminuria: a retrospective cohort study

**DOI:** 10.1038/s41598-023-43614-9

**Published:** 2023-09-29

**Authors:** Tatsuya Ishibashi, Shuhei Morita, Hiroto Furuta, Masahiro Nishi, Taka-Aki Matsuoka

**Affiliations:** 1 First Department of Medicine, Wakayama Medical University, 811‑1 Kimi‑idera, Wakayama City, Wakayama 641‑8509 Japan; 2 Department of Medical Technology, Faculty of Health Sciences, Kansai University of Health Sciences, Sennan, Osaka Japan

**Keywords:** Kidney diseases, Diabetes, Medical research

## Abstract

The renal protective effects of sodium-glucose co-transporter 2 (SGLT2) inhibitors and renin-angiotensin system (RAS) inhibitors on diabetic nephropathy without albuminuria have not been fully investigated. This retrospective cohort study focused on patients with type 2 diabetes mellitus who had a baseline estimated glomerular filtration rate (eGFR) of > 30 mL/min/1.73 m^2^, and a urinary albumin-to-creatinine ratio < 30 mg/gCr. After propensity score matching, using covariates such as age, body mass index, systolic blood pressure, hemoglobin A1c levels, and prescription history of RAS inhibitors, we established a cohort of 58 patients: the SGLT2 inhibitor group (n = 28) and the control group (n = 28). In this cohort, we compared the annual eGFR decline rate between the two groups. The SGLT2 inhibitor group exhibited a significantly smaller eGFR change than the control group (− 1.15 vs. − 2.18 mL/min/1.73 m^2^/year). Within the SGLT2 inhibitor group, patients prescribed RAS inhibitors had demonstrated an even smaller eGFR change (− 0.70 mL/min/1.73 m^2^/year). In conclusion, SGLT2 inhibitors also have safeguarding effects in the stage of diabetic nephropathy without albuminuria, and the combined use of a SGLT2 inhibitor and a RAS inhibitor appears to be more effective than the single use of each.

## Introduction

Diabetic nephropathy is the most common cause of chronic kidney disease (CKD). The typical clinical course of diabetic nephropathy is a gradual decline in glomerular filtration rate (GFR) with elevated proteinuria, leading to end-stage renal failure requiring hemodialysis or renal transplantation treatment.

In recent years, however, the clinical course of nephropathy in patients with diabetes has become more heterogeneous, reflecting an increase in the number of atypical patients whose renal function declines without overt albuminuria^1–3^. The occurrence of non-albuminuric diabetic nephropathy has several possible explanations, for example: coexisting vasculopathy or tubulointerstitial fibrosis, decreased estimated GFR (eGFR) due to previous episodes of acute kidney injury (AKI), or reduced albuminuria due to renin-angiotensin system (RAS) inhibitors^4^.

Angiotensin II causes constriction of the efferent arteriole of the renal glomeruli, which leads to an increase in intraglomerular pressure, and it causes glomerular hyperfiltration and increased proteinuria. The RAS inhibitors, angiotensin II type1 receptor blockers (ARBs) and angiotensin converting enzyme inhibitors (ACEIs), act protectively on the kidneys by inhibiting the action of angiotensin II. In a study of type 2 diabetic nephropathy, the use of losartan or irbesartan significantly reduced the composite endpoint of doubling serum creatinine levels, hemodialysis, and mortality^5,6^. Olmesartan treatment was also found to reduce the incidence of microalbuminuria in a study of normoalbuminuric patients with type 2 diabetes mellitus (T2DM) and cardiovascular risk (ROADMAP study)^7^. In another study in the early stage of diabetic nephropathy, telmisartan prevented the transition from micro-albuminuria to macro-albuminuria compared to a placebo, and telmisartan significantly reduced the onset of micro-albuminuria (INNOVATION study)^8^. Thus, RAS inhibitors are therefore known to attenuate the progression of diabetic nephropathy, irrespective of whether there is proteinuria.

In addition, sodium-glucose cotransporter-2 (SGLT2) inhibitors are hypoglycemic agents that promote urinary glucose excretion and have been reported to reduce the development of nephropathy as well as cardiovascular disease in several large clinical trials^9–11^. However, although SGLT2 inhibitors are definitely beneficial in suppressing the progression of diabetic nephropathy, it is still unclear whether they are also effective for patients with CKD who do not have albuminuria.

GFR generally declines at a rate of 1 mL/min/1.73 m^2^ per year, but the rate of decline is greater when renal function is impaired^12^. The risk of progression to end-stage renal disease (ESRD) increases in the order of overt proteinuria, microalbuminuria, and normal albuminuria, and the degree of urinary protein is associated with the degree of progression to renal failure. However, Krolewski et al. have reported that even in T2DM patients with normal renal function (eGFR > 60 mL/min/1.73 m^2^) and no proteinuria, there is a risk of progressive renal dysfunction, with an annual decline in eGFR of > 3 mlL/min occurring in 20% of patients^13^. Moreover, in a 12-year long-term follow-up study in patients with type 1 diabetes mellitus, early progressive renal function loss with eGFR exceeding -3.3% per year have occurred in 9% of the normoalbuminuria group and 31% of the microalbuminuria group^14^. These findings suggest the importance of assessing the rate of eGFR decline in an early stage.

In this study, we have evaluated the renal protective effects of SGLT2 inhibitors and RAS inhibitors on diabetic nephropathy without albuminuria according to the annual eGFR decline rate.

## Results

### Clinical characteristics at baseline

A total of 56 patients, 28 in the SGLT2 inhibitor group (Group S) and 28 in the control group (Group C), were assigned through propensity score matching. The patient backgrounds for both groups are shown in Table 1. Patients in both groups tended to be obese, with a mean body mass index (BMI) of 27.7 ± 4.9 in group S and 27.1 ± 3.7 in Group C. The two groups had no significant differences in blood pressure, hemoglobin A1c (HbA1c) levels, urine albumin-to-creatinine ratio (UACR), smoking status, and RAS inhibitor-prescription rates. Details of the SGLT2 and RAS inhibitors administered to patients in this study are shown in Table 2.

**Table 1:** Baseline characteristics of study participants (n = 56).

Characteristics	Total (n = 56)	Group C (n = 28)	Group S (n = 28)
Age (years)	59.4 (9.3)	58.3 (9.6)	60.5 (9.1)
Gender (Male/Female)*	34/22	15/14	19/8
BMI (kg/m^2^)	27.4 (4.3)	27.1 (3.7)	27.7 (4.9)
SBP (mmHg)	131.6 (15.3)	132.4 (15.4)	130.9 (15.5)
DBP (mmHg)	75.1 (10.6)	74.0 (9.6)	76.2 (11.5)
HbA1c (%)	7.5 (0.7)	7.6 (0.8)	7.4 (0.6)
eGFR (ml/min/1.73 m^2^)	77.5 (19.0)	77.7 (18.3)	77.3 (20.1)
UACR (mg/g･Cre)	11.7 (6.5)	11.2 (7.0)	12.2 (6.1)
Use of RAS inhibitors (%)			
None	28 (50.0)	14 (50.0)	14 (50.0)
ARB	26 (46.4)	13 (46.4)	13 (46.4)
ACEI	2 (3.6)	1 (3.6)	1 (3.6)
Smoking (%)			
Never	27 (48.2)	16 (57.1)	11 (39.3)
Former	20 (35.7)	7 (25.0)	13 (46.4)
Current	9 (16.1)	5 (17.9)	4 (14.3)

*BMI* body mass index, *SBP* systolic blood pressure, *DBP* diastolic blood pressure, *HbA1c* glycated hemoglobin, *UACR* urine albumin-to-creatinine ratio, *ARB* angiotensin II receptor blocker, *ACEI* angiotensin-converting enzyme inhibitor.

*The male/female ratio in each group was not significantly different, p = 0.15 on the chi-square test.

**Table 2:** Medications used in this study.

Medications	Total (n)	Group C	Group S
SGLT2i (n = 28)			
Empagliflozin	12	–	12
Ipragliflozin	5	–	5
Luseogliflozin	5	–	5
Canagliflozin	3	–	3
Tofogliflozin	3	–	3
ARB (n = 26)			
Telmisartan	9	3	6
Olmesartan	7	4	3
Candesartan	5	3	2
Valsartan	3	1	2
Irbesartan	2	2	0
ACEI (n = 2)			
Enarapril	2	1	1

*SGLT2i* sodium-glucose cotransporter 2 inhibitor, *ARB* angiotensin II type1 receptor blocker, *ACEI* angiotensin converting enzyme inhibitor.

### Outcomes

**Figure 1:**
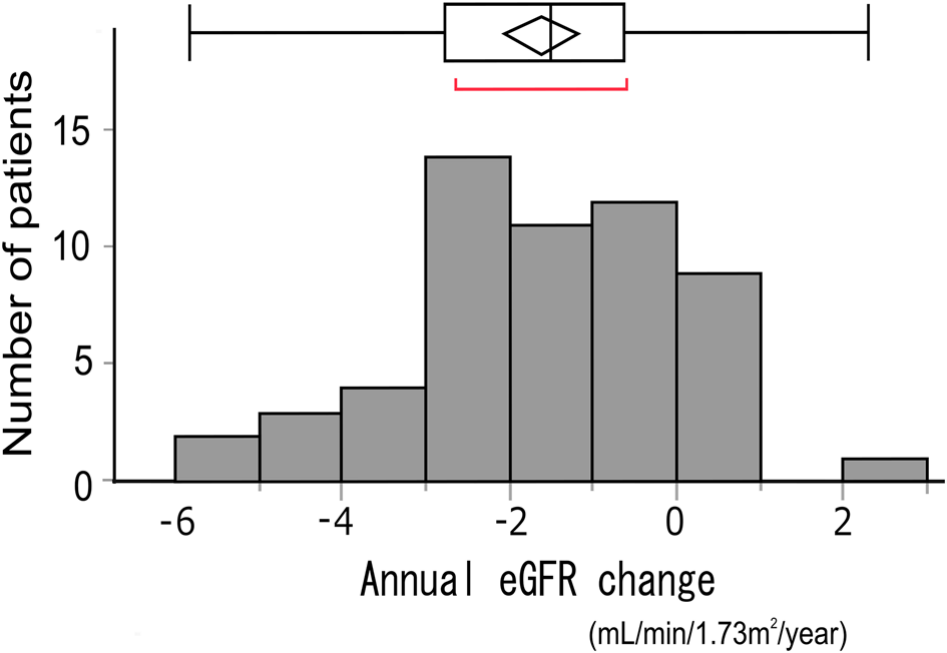
Frequency distribution of mean annual eGFR change in eligible patients. The population of eligible patients followed a normal distribution, Shapiro–Wilk normality test *p* = 0.733. The mean eGFR change was − 1.66 ± 1.64 mL/min/1.73 m^2^/year.

**Figure 2:**
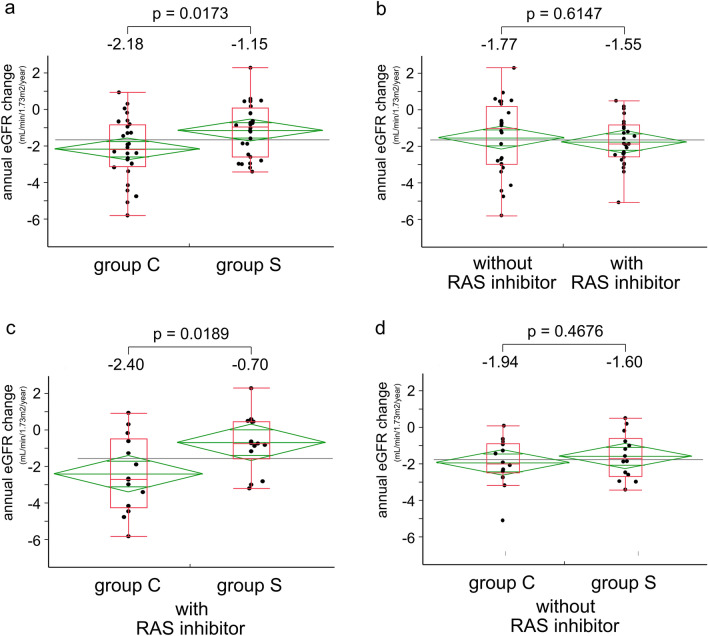
Mean annual eGFR change analyzed with and without SGLT2 and RAS inhibitors. The mean annual eGFR decline was smaller in the Group S than in the Group C (**a**), but there was no significant difference between the groups with and without a RAS inhibitor (**b**). The effect of SGLT2 inhibitors on the rate of eGFR decline was significant in the group with RAS inhibitors, whereas this effect was not observed in the group without RAS inhibitors (**c**,**d**).

The distribution of mean annual change of eGFR in the eligible patients is shown in Fig. 1. The mean eGFR change for the 58 eligible patients was − 1.66 ± 1.64 mL/min/1.73 m^2^/year. The mean annual change of eGFR in Group S was significantly smaller than that in Group C (− 1.15 ± 0.30 vs. − ﻿2.18 ± 0.30 mL/min/1.73 m^2^/year [*p* = 0.0173]) (Fig. 2a). Investigating the entire population divided according to being with and without prescription of RAS inhibitor, the change in mean eGFR was not significantly different. (*p* = 0.6147): − 1.55 ± 0.31 mL/min/1.73 m^2^/year in the group with RAS inhibitors and − 1.77 ± 0.31 mL/min/1.73 m^2^/year in the group without RAS inhibitors (Fig. 2b). Next, we examined the change in mean eGFR in Group S and Group C, stratified by the presence or absence of RAS inhibitors. The change in mean eGFR was not significantly different between the groups (Fig. 2c,d). It was − 0.70 ± 0.48 (Group S) and − 2.40 ± 0.48 (Group C) in the group receiving a RAS inhibitor (*p* = 0.0189), while in the group not receiving a RAS inhibitor, the change was − 1.60 ± 0.34 (Group S) and − 1.95 ± 0.34 (Group C).

## Discussion

Previous reports on the renoprotective effect of SGLT2 inhibitors have mainly analyzed the combined renal endpoints, including end-stage renal failure, creatinine doubling and death from renal disease, or a decrease in urinary albumin. Conversely, there has not been adequate investigation of the renal protective effect on the stage of diabetic nephropathy without albuminuria. The annual eGFR decline rate has recently been considered to be a useful surrogate clinical marker for kidney disease progression^15^. In this study, we therefore evaluated the effect of RAS inhibitors and SGLT2 inhibitors with the annual eGFR decline rate as an evaluation index on patients with T2DM without albuminuria.

First, we observed that SGLT2 inhibitors also had safeguarding effects in diabetic nephropathy without albuminuria. The renoprotective effects of SGLT2 inhibitors are multifaceted. One of the mechanisms involves reducing sodium reabsorption in the proximal tubule, which activates the tubulo-glomerular feedback (TGF) mechanism. This activation occurs due to an increased influx of Na+ at the juxtaglomerular apparatus, leading to the constriction of the afferent arteriole and, ultimately, resulting in a decrease in the glomerular capillary hypertension and filtration^16^. Other mechanisms by which SGLT2 inhibitors exert their renoprotective effects have been reported, including decreased ATP energy consumption in the renal tubular epithelium due to sodium-hydrogen exchanger (NHE)3 inhibition and improved vascular endothelial cell function due to reduced reactive oxygen species (ROS) production in the mitochondria^17–19^.

SGLT2 inhibitors not only directly impact upon various components of the kidney, they also affect on renal protection through cardiorenal interactions because of the close relation between heart failure and renal failure. A decline in renal function leads to the retention of volume, contributing to the development of heart failure. Conversely, reduced cardiac function and consequent reduced renal perfusion pressure tend to activate the sympathetic nervous system (SNS), which in turn promotes renin–angiotensin–aldosterone system (RAAS)^20–23^.

Since SGLT2 inhibitors have protective effects on the heart and the kidneys, so mutually protective effects on both organs can be expected. SGLT2 inhibitors have been shown in several large clinical trials to have positive impacts on outcomes in both heart failure and CKD^9–11^. Notably, their efficacy has been demonstrated even in patients with comorbidities such as frailty, and SGLT2 inhibitors have shown promising results in improving markers of AKI in patients with acute heart failure^17,24^.

**Figure 3:**
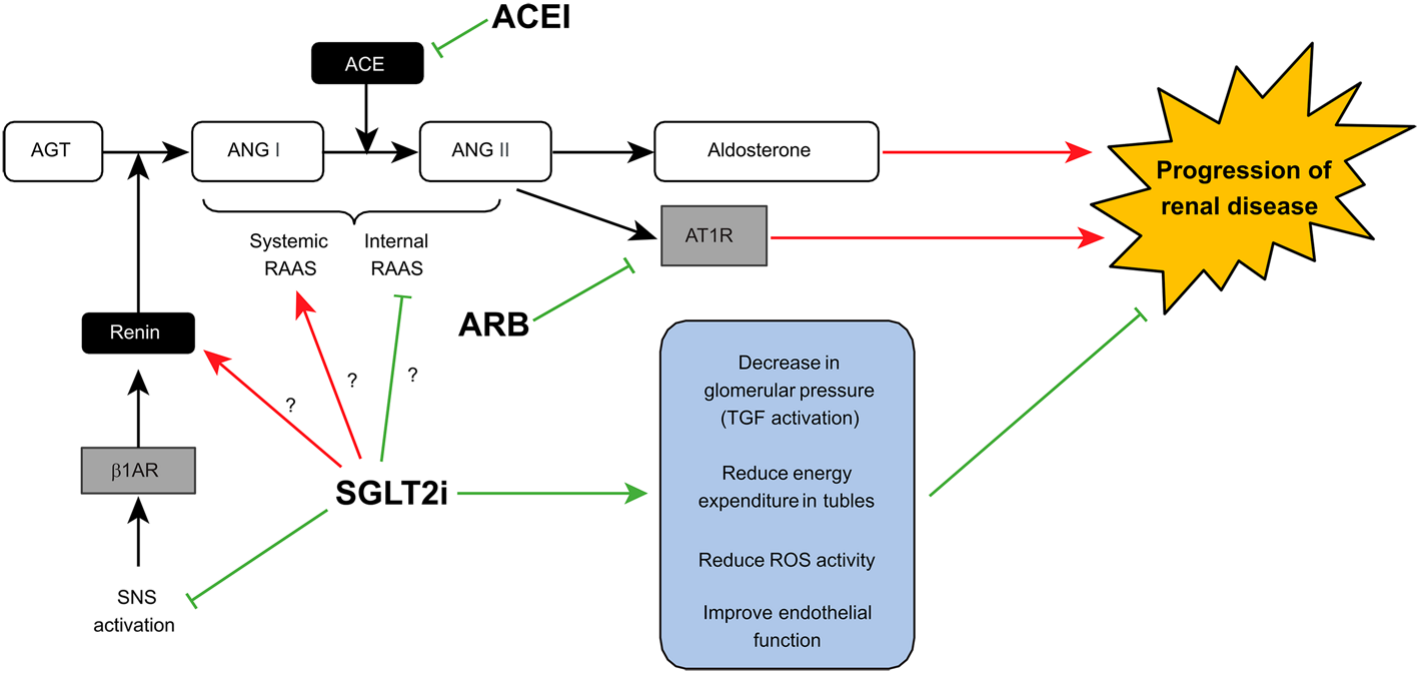
Dual benefits of the SGLT-2 inhibitor and RAS inhibitor pathways on renal outcomes. AGT: angiotensinogen; ANG I: angiotensin I; ANG II: angiotensin II; ACE: angiotensin converting enzyme; ACEI: angiotensin converting enzyme inhibitor; RAAS: renin–angiotensin–aldosterone system; ARB: angiotensin II receptor blocker; AT1R: angiotensin II receptor type 1; SGLT2i: sodium-glucose cotransporter-2 inhibitor; TGF: tubulo-glomerular feedback; β1AR: β1-Adrenergic Receptor; SNS: sympathetic nervous system; ROS: reactive oxygen species.

In our study, a robust renoprotective effect was observed when RAS inhibitors were combined with SGLT2 inhibitors in patients with normoalbuminuria. This finding may indicate a synergistic effect of the two agents. ARBs bind to angiotensin II type 1 receptors, blocking the physiological effects of angiotensin II, while ACEIs inhibit ACE activity, thereby reducing angiotensin II production. Although their mechanisms are not exactly the same, both ACEIs and ARBs, which are classified as RAS inhibitors, have shown effectiveness in reducing albuminuria and slowing the decline in renal function in patients with CKD, mainly due to improvement of glomerular hyperfiltration. The effects of SGLT2 inhibitors on RAAS have been widely reported. In the acute phase after initiation of SGLT2 inhibitors, diuresis and hemodynamic effects increase RAAS activity. In the chronic phase, some reports suggest that the internal RAAS in the proximal tubules is decreased, but the systemic RAS is maintained or enhanced^25^. Moreover, SGLT2 inhibitors act at multiple points to modulate SNS activity, which leads the improvement of renin secretion activated through β1-adrenergic receptors^26,27^. This is particularly crucial in cases of heart failure with preserved ejection fraction (HFpEF), where both the RAAS and SNS are concurrently activated^28^. The cooperative intensified RAAS inhibition by the combination therapy seems to be one of the mechanisms contributing to the renoprotection. Alternatively, it is possible to think that multifaceted effects of the SGLT2 inhibitor still requires further RAAS inhibition for the renoprotection from the stage of normoalbuminuria (Fig. 3).

Renoprotective effect has been reported in other drugs for diabetes. Han et al. reported that thiazolidinediones exhibited an effect on reducing the progression of renal complications in mouse models of obesity and T2DM, especially in combination with SGLT2 inhibitors. This combination treatment also suppressed angiotensinogen mRNA expression in the renal cortex, along with a reduction in inflammatory and fibrosis markers^29^. In addition, GLP-1 receptor agonists (GLP-1RAs) have been shown to offer renal protection in patients with diabetes^30^. While the precise mechanism remains incompletely understood, but GLP-1 and its agonists appear to reduce water and sodium reabsorption in the proximal tubule by modulating the RAAS through the suppression of renin secretion, lowering of ANG II levels, and inhibition of NHE3^30^. In this study, thiazolidinediones and GLP-1RA were prescribed to a small number of patients, but as there was no significant difference in prescription rates between both groups, we suggest that it is unlikely to have impacted the results.

The mean eGFR at the beginning of observation was 78 mL/min/1.73 m^2^ (Supplementary data 1) and among the 11 patients who had an average eGFR decline rate of 3.0 mL/min/1.73 m^2^ or more per year (4 patients in Group S, and 7 patients in Group C), all had an eGFR of 70 mL/min/1.73 m^2^ or higher at the beginning of observation (Supplementary data 2). This suggests that even patients who do not meet CKD criteria can have a relatively rapid decline in renal function. To reduce the risk of adverse renal outcomes, it is therefore crucial to monitor changes in eGFR over time and to proceed with early intervention, such as by using SGLT2 inhibitors in combination with RAS inhibitors or by adjusting lifestyle.

Finally, this study has several limitations. First, this is a retrospective observational study and presents exploratory findings based on a limited patient sample. Furthermore, despite the use of propensity scores to adjust for covariates, there remains the possibility of residual confounding by unmeasured factors. The values of covariates used are data from the start of the observation, so we exclude the possibility that the change of confounders during the follow-up period could have affected the result.

In conclusion, in normoalbuminuric patients with T2DM, SGLT2 inhibitor offered greater protection against decreased renal function when used in combination with RAS inhibitors. Concomitant use of SGLT inhibitors and RAS inhibitors is suggested to be important for preserving renal function even in normoalbuminuric patients with T2DM.

## Materials and methods

### Study design and patient selection

The eligible subjects for this study were patients with T2DM who visited the First Department of Medicine at Wakayama Medical University between January 2017 and December 2021, and met all of the following criteria: aged 20 years or older, prescribed one or more medications for diabetes, had a baseline eGFR of 30 mL/min/1.73 m^2^ or higher, had a baseline UACR of 30 mg/gCr or less, and eGFR was measured at least four times per year for five consecutive years from the beginning of the study.

Excluded from this study wore patients with type 1 diabetes mellitus, pancreatic diabetes, steroid-induced diabetes, a history of initiation, interruption, or discontinuation of SGLT2 inhibitors and RAS inhibitors (ACEIs or ARBs) during the observation period, as well as patients who had taken mineralocorticoid receptor antagonists or direct renin inhibitors.

**Figure 4:**
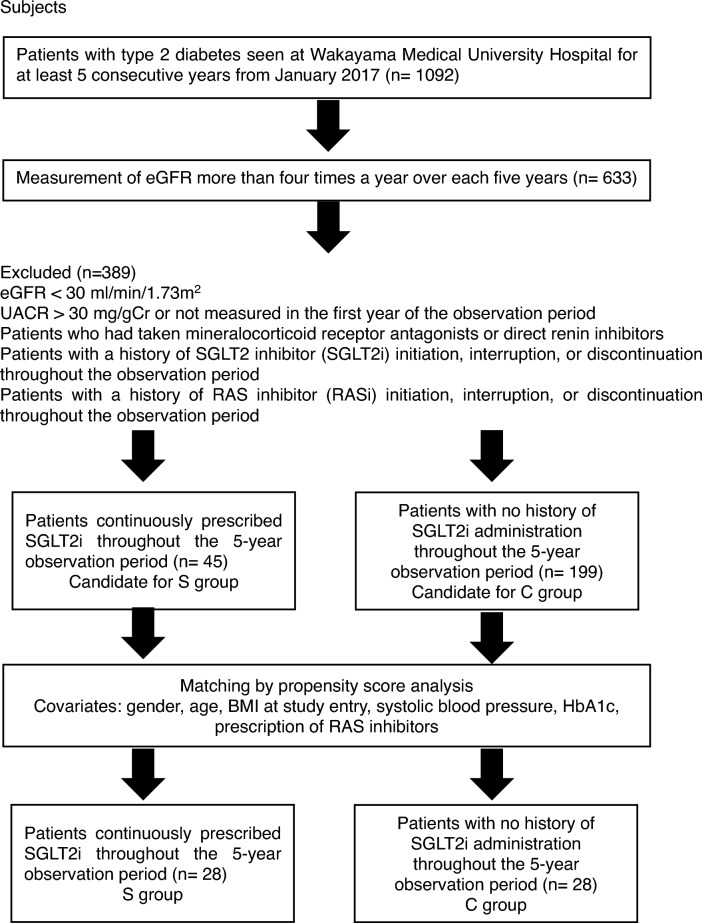
Flow chart of the study.

Among the eligible patients, 45 patients who were continuously prescribed SGLT2 inhibitors during the 5-year observation period and 199 patients who had never received SGLT2 inhibitors were selected, and matched by propensity score analysis using sex, age, BMI at the start of the study, systolic blood pressure, HbA1c levels, and prescription of RAS inhibitors as covariates. Twenty-eight patients in the Group S and 28 patients in the Group C were selected for analysis. The mean annual eGFR decline rate was analyzed retrospectively for both groups, with and without RAS inhibitors (Fig. 4).

This study was conducted with the approval of the Wakayama Medical University Research Ethics Committee and in compliance with the Declaration of Helsinki, and it involved retrospective research with informed consent obtained through an opt-out method.

### Definition of T2DM

T2DM in this study was defined as a clinical diagnosis following Japan Diabetes Society (JDS) guidelines. It excluded those with type 1 diabetes mellitus, cirrhosis, a history of pancreatic surgery, or the use of steroid medication^31^.

### Definition of RAS inhibitors

This study defines ARBs and ACEIs as RAS inhibitors. Subjects who were prescribed direct renin inhibitors or anti-aldosterone drugs were not included.

### Definition of clinical parameters

Age was defined as the patient’s age at the beginning of the observation period.

BMI, systolic blood pressure, and HbA1c levels were obtained from the records of the patient’s first outpatient visit in the year the observation period started.

HbA1c levels were expressed according to the National Glyco-hemoglobin Standardization Program (NGSP) guidelines (%).

Albuminuria levels were classified based on the spot UACR at the first year of follow-up, and normal albuminuria was defined as UACR < 30 mg/gCr according to the 2012 KDIGO CKD guidelines^32^.

### Calculation of estimated GFR (eGFR) and the annual eGFR decline rate

eGFR was calculated using the equation formula developed by Matsuo et al. for Japanese individuals, which considers age, sex, and serum creatinine levels^33^.

Administration of SGLT2 inhibitors is well known to potentially cause a temporary decrease in eGFR. To minimize this effect, eGFR data obtained during the first 90 days after initiation of SGLT2 inhibitors were excluded from this study.

The eGFR is calculated based on serum creatinine measurement, which can vary due to measurement conditions. To minimize the effect of this variation, the average eGFR for each year was obtained for each case, and the annual eGFR decline rate was calculated based on the slope of the linear approximation.

### Statistical methods

To minimize bias for potential confounders, SGLT2 inhibitor-treated and non-treated groups were selected using propensity scores calculated by logistic regression using the baseline characteristics described above. A greedy nearest neighbor matching algorithm with a caliper width equal to 0.2 of the logit standard deviation of the propensity score was used to create matched pairs between the SGLT2 inhibitor-treated and non-treated groups. The equilibrium of the matching model was assessed using standardized differences in the means of each covariate. A threshold of 0.20 was used to identify imbalance in the baseline covariates. Matching model refinements were repeated until balance was achieved.

Data are presented as mean ± standard deviation, and Student's t-test was used to compare means between the two groups. The significance level for each test was defined as *p* < 0.05. All analyses were conducted using JMP software (JMP Pro 16.0.0; SAS Institute, Japan). The normality of distribution was checked using the Shapiro–Wilk normality test, followed by comparison with an unpaired Student's t-test.

### Supplementary Information

Supplementary Information 1.

Supplementary Information 2.

## Data Availability

The datasets used and/or analyzed during the current study are available from the corresponding author upon reasonable request.
