# Supplementary material for: Renoprotective potential of concomittant medications with SGLT2 inhibitors and renin-angiotensin system inhibitors in diabetic nephropathy without albuminuria: a retrospective cohort study
Source: Sci Rep. 2023 Sep 29;13:16373. doi: 10.1038/s41598-023-43614-9 (PMC10541410; doi:10.1038/s41598-023-43614-9)
Supplement: Supplementary file 1 — Supplementary Information 1. [file 41598_2023_43614_MOESM1_ESM.pdf]

Supplementary data 1

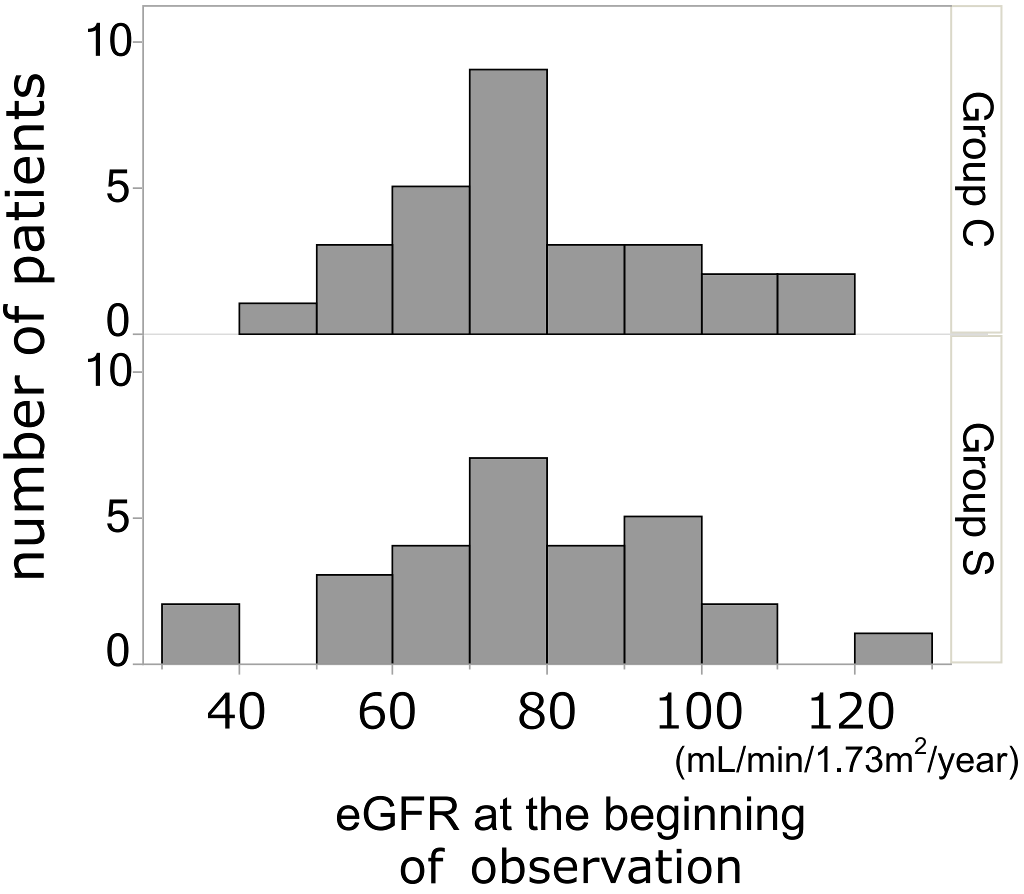

Supplementary data 1
eGFR at the start of observation in the target patients
The eGFR of the matched group S and group C eligible patients followed a normal distribution, with most having an eGFR of 60 mL/min/1.73m^2 or higher.
