# Supplementary material for: Renoprotective potential of concomittant medications with SGLT2 inhibitors and renin-angiotensin system inhibitors in diabetic nephropathy without albuminuria: a retrospective cohort study
Source: Sci Rep. 2023 Sep 29;13:16373. doi: 10.1038/s41598-023-43614-9 (PMC10541410; doi:10.1038/s41598-023-43614-9)
Supplement: Supplementary file 2 — Supplementary Information 2. [file 41598_2023_43614_MOESM2_ESM.pdf]

Supplementary data 2

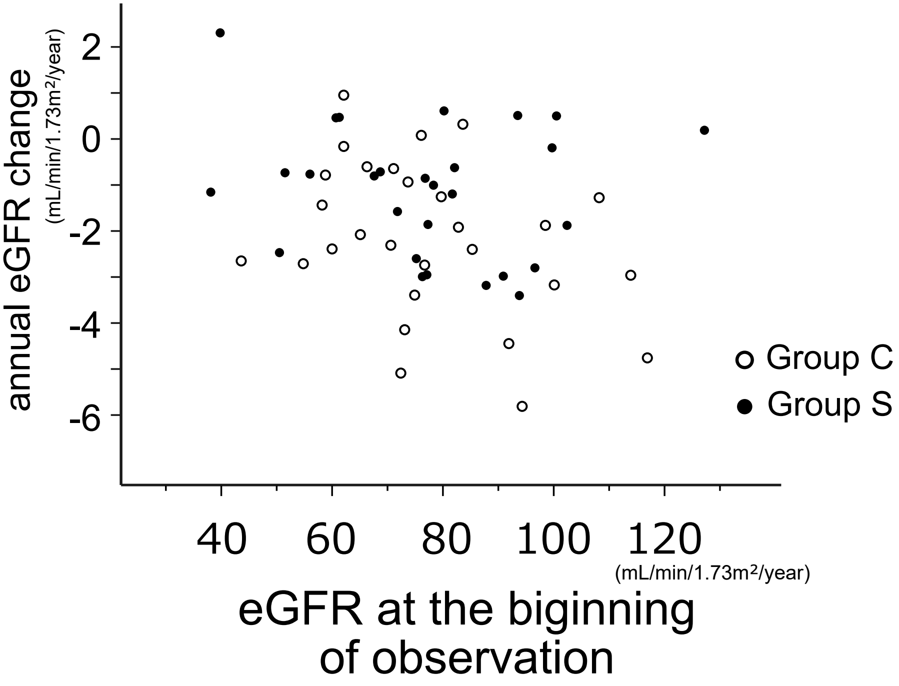

Supplementary data 2
Scatterplot of eGFR at start and rate of decline of eGFR by group S (filled circles) and group C (open circles). There was no significant correlation between eGFR at the start and the rate of eGFR decline, but eGFR declined by more than 3 per year only in those cases in which the eGFR at the start of observation remained above 70 mL/min/1.73m^2.
